# Méningo-encéphalite à Streptococcus agalactiae chez l'adulte non immunodéprimé

**DOI:** 10.11604/pamj.2015.20.5.4526

**Published:** 2015-01-05

**Authors:** Mostafa Rafai, Naoufal Chouaib, Saad Zidouh, Hicham Bakkali, Lahcen Belyamani

**Affiliations:** 1 Pôle des Urgences Médico-chirurgicales de l'hôpital militaire d'instruction Mohammed V, Rabat, Maroc

**Keywords:** Méningo-encéphalite, infection, streptococcus agalactiae, Meningoencephalitis, infection, streptococcus agalactiae

## Abstract

*Streptococcus agalactiae* est un Streptocoque beta-hémolytique du groupe B (SGB), c'est un germe commensal occasionnel de la peau, du tube digestif et des voies génito-urinaires. Nous rapportons un cas inhabituel d'une méningo-encéphalite due au *Streptococcus agalactiae* (SGB) multisensible à l'antibiogramme chez un sujet adulte immunocompétent admis au service des urgences pour prise en charge de troubles de conscience fébrile. L’évolution clinique et biologique à J10 était favorable et le patient à été transféré au service de neurologie pour complément de prise en charge secondaire. L'originalité de notre observation réside dans la rareté du type d'infection par ce germe puise qu'elle est la troisième à notre connaissance d'une méningo-encéphalite à *Streptococcus agalactiae* dans la littérature, c'est ainsi que même s'il est très rarement en cause, il doit être considéré comme une étiologie possible de méningo-encéphalite chez l'adulte en dehors de la grossesse, quelle que soit le statut immunitaire du patient, et sans méconnaitre le rôle du terrain sous-jacent dans l’émergence de cette pathologie infectieuse polymorphe est potentiellement grave.

## Introduction

*Streptococcus agalactiae* ou streptocoque du groupe B (SGB) est connu pour son implication dans certaines infections graves du nouveau-né et de la femme enceinte [1], et est classiquement considéré comme un germe rarement impliqué dans la pathologie infectieuse de l'adulte à l'exception des infections du tractus urinaire chez les femmes enceinte. L'attention a été portée ces derniers temps sur d'autres infections à SGB survenant chez l'adulte, notamment des infections de la peau et des tissus mous, des bactériémies, des pneumopathies, des méningites, des endocardites, des péritonites et des infections ostéo-articulaires [2]. *Streptococcus agalactiae* est une cause importante d'infections invasives graves, fréquemment mortelles chez les adultes en dehors de la grossesse, touchant plus particulièrement les personnes âgées ayant des facteurs favorisants sous-jacents [1]. Nous rapportons un cas inhabituel d'une méningo-encéphalite (ME) due au *Streptococcus agalactiae* (SGB) chez un sujet adulte immunocompétent.

## Patient et observation

Un patient âgé de 70 ans, ayant comme antécédents une hypertension artérielle et un diabète équilibrés, était admis au service des urgences pour prise en charge d'un trouble de conscience fébrile évoluant depuis 24 heures. À l'admission, il était fébrile à 39,7°C, inconscient avec un score Glasgow Coma Scale (GCS) à 11/15 et sans signes de focalisation ni convulsions, une fréquence respiratoire à 22 cpm, une saturation pulsée en oxygène à 95% à l'air ambiant, passé à 100% sous masque d'O^2^, une pression artérielle à 142/93 mm Hg et un pouls à 105 bpm. L'examen clinique trouvait une raideur de la nuque, alors que le reste de l'examen était sans anomalies (notamment l'absence de purpura fulminans). Devant ce tableau clinique une méningo-encéphalite infectieuse à été suspecté; le bilan biologique à révélé des globules blancs(GB) à 18800/mm^3^, une C-reactive protein(CRP) à 242 mg/l et un bilan d'hémostase normal. La tomodensitométrie (TDM) cérébrale était sans anomalies. Le patient à bénéficié après d'une ponction lombaire qui à révélé à l'examen cytobactériologique des hématies à 500/mm^3^, des leucocytes à 400/mm^3^ avec une formule panachée, et la présence de Cocci à Gram positif à l'examen direct. La biochimie du LCR à montré une protéinorachie à 5,82 g/l et un rapport glycorachie/glycémie à 0,25. Une antibiothérapie à été immédiatement mise en route à base de céfotaxime associée à une corticothérapie, le dexaméthasone, la culture confirmait la présence de nombreux Cocci à Gram positif et identifiait un Streptococcus du groupe B(SGB) ou *Streptococcus agalactiae* sensible à tous les antibiotiques notamment la céfotaxime et exception faite du sulfaméthaxazole + triméthoprime; le reste du bilan biologique revenait sans anomalies notamment les sérologies de syphilis, HIV, HVB, HVC qui étaient négatives et les CD4 et CD8 sans anomalies. Un électroencéphalogramme (EEG) réalisé 24 heures après concluait à un ralentissement de l'activité de fond bihémesphérique, L’évolution après 48 heures était marquée sur le plan clinique par une nette amélioration de l’état de conscience avec un GCS à 14/15, ainsi qu'une bonne évolution biologique notamment sur le liquide de ponction lombaire de contrôle et la CRP. À J10, l’évolution était marquée par la récupération d'un état de conscience normal avec un GCS à 15/15, et un LCR stérile; à l'issue de cette évolution favorable, le patient à été transféré au service de neurologie pour éventuel EEG de contrôle et complément de prise en charge secondaire.

## Discussion

*Streptococcus agalactiae* est un Streptocoque beta-hémolytique du groupe B (SGB), c'est un germe commensal occasionnel de la peau, du tube digestif et des voies génito-urinaires (portage vaginal estimé à 40%) [3], et qui était habituellement considéré comme un agent pathogène touchant principalement les nouveau-nés et les femmes en péri-partum. Les SGB sont classés en différents sérotypes correspondant à des différences structurales des polysaccharides capsulaires (Ia, Ib, and II-VII) et la présence ou l'absence de protéines antigéniques de surface. Des recherches ont été réalisées pour identifier les souches de SGB impliquées dans les infections de l'adulte en dehors de la grossesse, une étude-clé a montré une prédominance des sérotypes capsulaires Ia, III, et V [4], alors que Le sérotype II est le plus souvent incrimine dans les atteintes infectieuses méningées de l'adulte [5]. Les étapes du processus infectieux sont peu connues. Ces bactéries adhèrent aux surfaces de différents types de cellules épithéliales grâce à leur protéine alpha C. Les cellules épithéliales sont ensuite envahies, ouvrant la voie à la dissémination systémique [6]. Plusieurs facteurs de virulence, comme la capsule polysaccharidique et l'hémolysine, permettraient aux souches d’échapper au système immunitaire de l'hôte [7]. Le rôle d'autres produits bactériens, comme la superoxide dismutase et l'acide D-alanyl-lipotéichoique a été évoqué [6].

Néanmoins, l'incidence des infections graves par le SGB a augmenté au cours de ces dernières années chez les adultes en dehors de tout contexte de grossesse, suggérant une modification du « spectre » des infections à ce germe [2]; Les raisons de l'augmentation de l'incidence des infections à SGB chez l'adulte ne sont pas précisément connues mais elle pourrait être expliquée par une plus grande prévalence des souches virulentes ou par un accroissement du nombre de patients ayant des facteurs favorisants [2]; Ainsi, parmi les principaux facteurs favorisants démontrés on rapporte l’âge (facteur indépendant d'infection), le diabète (présent chez 20 à 25%), le cancer du sein, la cirrhose hépatique, l'insuffisance rénale, l'insuffisance cardiaque, l'impossibilité de se lever du lit ou un antécédent d'accident vasculaire cérébral [7]. Au moins un facteur est présent dans plus de 90% des cas [7]; c'est ainsi que le risque pour un patient diabétique ou atteint d'une néoplasie de développer une infection à SGB est respectivement dix et seize fois plus élevé que pour la population témoin [1], comme c'est le cas chez notre patient diabétique et âgé de 72ans. Parmi ces infections invasives touchant l'adulte, il y a les infections neuroméningées, les méningites à SGB sont très rares chez l'adulte mais la mortalité est élevée (34%) proche de la mortalité observée avec le pneumocoque [8]; quant aux méningo-encéphalites elles demeurent extrêmement rares. En faite et à notre connaissance, seulement deux observations de méningo-encéphalites à SGB ont étés rapportées dans la littérature, la première par Hoen [9] en 1993, et la deuxième par Rbibaro [10] en 1998.

Les méningo-encéphalites sont des maladies plutôt rares caractérisées par un processus inflammatoire touchant le tissu cérébral associé à une réaction méningée, La physiopathologie des ME infectieuses est encore incomplètement comprise, Les lésions cérébrales sont provoquées par l′invasion et la réplication de l′agent infectieux au sein du parenchyme cérébral [11]. Le diagnostic est suspecté devant l'association d'une méningite (syndrome méningé fébrile, hypercellularité du liquide céphalorachidien) et de signes cliniques traduisant une souffrance encéphalitique: confusion, désorientation, troubles du comportement, signe(s) de localisation, épilepsie [12]; en faite, la clinique des méningo-encéphalites à *Streptococcus agalactiae* n'a pas de signes spécifiques et la biologie du liquide céphalorachidien est classiquement celle d'une méningite bactérienne, avec hypercellularité à prédominance de polynucléaires neutrophiles, une hyperprotéinorachie et une hypoglycorachie; le point d'entrée du micro-organisme est soit l'altération de la barrière mécanique soit la dissémination hématogène. *S. agalactiae*reste sensible aux pénicillines, aux céphalosporines et à la vancomycine [13]. Les pénicillines sont les antibiotiques de choix pour le traitement et la synergie avec les aminoglycosides est préservée [1]. Le taux de mortalité pour les personnes adultes est plus élevé que dans le cas des infections du jeune enfant ou de la femme enceinte (environ 15% versus 5%), la moitié de la mortalité attribuable au *S. agalactiae*survenant chez les patients de plus de 65 ans [13].

## Conclusion

Nous rapportons un cas inhabituel d'une infection invasive à *Streptococcus agalactiae*: une méningo-encéphalite infectieuse chez un adulte non immunodéprimé avec une bonne évolution. L'originalité de notre observation réside dans l'extrême rareté de la localisation de l'infection due à ce germe, puise qu'elle est la troisième à notre connaissance dans la littérature d'une méningo-encéphalite à *Streptococcus agalactiae*après celle de Hoen [9] publiée en 1993 et celle de Robibaro [10] publiée en 1998, ainsi que dans l’évolution rapidement favorable avec récupération neurologique ad integrum et sans séquelles. C'est ainsi que même s'il est très rarement en cause, le *Streptococcus agalactiae* doit être considéré comme une étiologie possible de méningo-encéphalite infectieuse chez l'adulte en dehors de la grossesse, quelle que soit le statut immunitaire du patient, et sans méconnaitre le rôle du terrain sous-jacent dans l’émergence de cette pathologie infectieuse polymorphe est potentiellement grave.
